# The effects of smoking and hypertensive disorders on fetal growth

**DOI:** 10.1186/1471-2393-6-16

**Published:** 2006-04-21

**Authors:** Svein Rasmussen, Lorentz M Irgens

**Affiliations:** 1 Medical Birth Registry of Norway, Locus of Registry Based Epidemiology, University of Bergen and the Norwegian Institute of Public Health, Bergen, Norway; 2 Institute of Clinical Medicine, Department of Obstetrics and Gynecology, University of Bergen, Bergen, Norway

## Abstract

**Background:**

It is well known that smoking and pregnancy induced hypertension (PIH) are associated with decreased fetal growth. It has been reported that in preeclampsia the fetal growth deficit attributable to smoking is higher, which has been contradicted in other studies. We therefore evaluated the effects on fetal growth of early- and late onset PIH and chronic hypertension and how cigarette smoking modify these effects. We also quantified the proportion of small for gestational age (SGA) cases attributable to PIH, chronic hypertension, and smoking.

**Methods:**

Population-based study based on record of 215598 singleton pregnancies from the Medical Birth Registry of Norway.

**Results:**

In severe preeclampsia, mild preeclampsia, transient hypertension, and normotension in term birth, odds ratios (ORs) of SGA in smokers compared with non-smokers were 1.4 (95% confidence interval 0.9, 2.2), 1.6 (1.3, 1.9), 2.3 (1.8, 3.1), and 2.0 (1.9, 2.1), respectively. For preterm births, corresponding ORs were 1.3 (0.9, 2.0), 1.8 (1.1, 3.0), 4.1 (1.9, 9.0), and 1.7 (1.4, 2.0), respectively. The effect of early onset PIH was stronger than that in term births, while the effect of smoking was equal in preterm and term newborns. Only in non-smokers who delivered at term, the rates of SGA significantly increased with the severity of PIH (ORs = 1.3 (1.1, 1.5), 1.8 (1.7, 2.0), and 2.5 (2.2, 3.0) for transient hypertension, mild-, and severe preeclampsia, respectively). The combined effects of smoking and hypertension were generally not synergistic. The effect of smoking was not stronger in women who had chronic hypertension. Nor were the effects of chronic hypertension stronger in smokers. PIH explained 21.9 and 2.5% of preterm and term cases of SGA, respectively, while smoking explained 12% of SGA cases.

**Conclusion:**

The effects of hypertensive disorder and smoking were generally not synergistic, which suggest that they may exert their main actions on separate sites or work through separate mechanisms within or outside the placenta. If smoking were eliminated in pregnant women, the number of SGA cases would be reduced by 12%.

## Background

The hypothesis that placental dysfunction is involved in the development of pregnancy induced hypertension (PIH) has been supported by numerous studies [1-3]. Thus, clinical studies have suggested that preclampsia, especially early onset, often is preceded by placental dysfunction in terms of fetal growth restriction (FGR) [4]. Shallow invasion by fetal trophoblasts in maternal spiral arteries in early pregnancy, which may cause occlusion of the vessels, has been observed both in preeclampsia and FGR [1,2]. Eventually, products released by the ischemic placenta cause endothelial activation resulting in hypertension and proteinuria as well as decreased fetal growth [5]. Still, the pathogenesis of preeclampsia is far from clarified and it has even been suggested that early and late onset preeclampsia represent two different diseases [6,7]

It is well known that also cigarette smoking is associated with FGR. On the other hand, many studies have reported that smoking reduces the risk of PIH [8-10]. The effects on fetal growth of smoking in women who have PIH and, *vice versa*, of PIH in women who smoke, are unclear. Neither is it known whether such effects are different in early and late onset PIH. It has been reported that in preeclampsia the fetal growth deficit attributable to smoking is higher [9], which has been contradicted in other studies [11-13]. Furthermore, we are not aware of studies that have explored the combined effects of smoking in the different subgroups of hypertensive complications in pregnancy.

In the present study we wanted to evaluate and compare the effects on fetal growth of early- and late onset PIH (transient hypertension, mild preeclampsia, and severe preeclampsia) as well as chronic hypertension and how cigarette smoking modify these effects. We also wanted to quantify the proportion of SGA cases attributable to PIH, chronic hypertension, and smoking.

## Methods

Since 1967, all births in Norway are notified to the Medical Birth Registry of Norway based on compulsory notification [14]. More than 99% of pregnant women receive standardized antenatal care [15]. The registry comprises medical data on all live births and abortions at 12 weeks gestation or more including abortions induced on medical indications. Data are transferred by the midwives to the notification form from the pregnancy record which the women bring to the delivery unit. Within the ninth day post partum, the notification form is completed and sent to the Medical Birth Registry. In 1999, a revised version of the notification form was implemented to include new variables like data on maternal smoking habits. Subgroups of hypertensive disorders in pregnancy are notified by checking of boxes [14].

The present study was based on records of all single births in Norway from 1999 to 2002, altogether 223541 births. We excluded pregnancies with gestational age outside the range 27–42 weeks of gestation (*n *= 5294), women with chronic renal disease (*n *= 918), rheumatoid arthritis (*n *= 270), heart disease (*n *= 1146), and diabetes mellitus (*n *= 1235), leaving 215598 pregnancies for study. Gestational age was based on ultrasound scanning at 17–19 weeks of gestation. In pregnancies lacking data on ultrasound dating (3%), gestational age was based on the last menstrual period.

Clinical criteria of PIH in Norway have been in accordance with the recommendations by the American College of Obstetricians and Gynecologists in 1972 [16], which are also referred to in the Medical Birth Registry's instructions for completion of the notification form. Transient hypertension implies pregnancy induced hypertension without proteinuria with BP ≥ 140/90 (one or both values exceeded) or rise in systolic BP ≥ 30 mmHg or diastolic BP ≥ 15 mmHg after 20 weeks of gestation. Mild preeclampsia implies systolic BP 140–159, diastolic BP 90–109, rise in systolic BP ≥ 30 mmHg, or diastolic BP ≥ 15 mmHg and proteinuria ≥ 1+. Severe preeclampsia implies BP ≥ 160/110 and/or proteinuria ≥ 2+.

Daily smokers and non-smokers at the beginning of pregnancy were followed to delivery with respect to the size of their newborns. The two cohorts were divided into six mutually exclusive groups: transient hypertension, mild and severe preeclampsia, chronic hypertension without preeclampsia, preeclampsia superimposed on chronic hypertension, and normotensive pregnancies (**Tables 1–6 **[see Additional file 1] [see Additional file 2]).

### Statistical analysis

To achieve normal distributions, birthweight was ln-transformed. Birthweight was regressed against gestational age using fractional polynomials [17]. In order to calculate gender and birth order specific birthweight percentiles, birth order (1 or 2+) and fetal gender were added to the polynomial equation. The method of scaled absolute residuals was used to model standard deviation (SD) against gestational age [18]. For each observation, the standard deviation score (z-score) was the distance in SDs from the mean regression line. The 10^th ^birthweight percentile was calculated as mean – 1.282 SD.

The associations of PIH and chronic hypertension with SGA were estimated by odds ratios (ORs) obtained from logistic regression analysis, in which we adjusted for maternal age in years (19 or less, 20–29, 30–34, and 35 or more). Effects of hypertension on birthweight and its SD score were also assessed by analysis of variance were we adjusted for maternal age. Birthweight deficits attributable to smoking and hypertensive disorder, as expressed as mean reduction in SD scores of birthweight, were calculated by post hoc pairwise comparisons. To investigate modifications of the effects on birthweight of smoking and hypertension, we included a multiplicative interaction term of smoking with each five hypertensive disorders in logistic regression models. A non-significant interaction term indicated that smoking and e.g. severe preeclampsia did not affect each other. An interaction term in which the odds ratio (OR) was significantly below 1 was considered antagonistic, while an interaction term in which the OR significantly exceeded 1 was considered synergistic [19].

To estimate the proportion of all cases of SGA attributable to hypertensive disorder and smoking, adjusted population attributable risk fractions (PAR%) were calculated as proportion of exposed among all cases × (RR-1)/RR, where RR is the adjusted relative risk [20]. ORs adjusted for smoking and maternal age were used to calculate approximate adjusted RRs [21].

We used SPSS (Statistical Package for the Social Sciences; SPSS, Inc, Chicago, IL) for analysis.

We confirm that research ethics committees in Norway regularly exempt research on anonymized registry data from ethical review.

## Results

Women with chronic hypertension were older than those who were normotensive or had PIH and women with transient hypertension were older than those who had preeclampsia (**Table 1 **[see Additional file 1]). Compared with normotensive women, those with PIH and superimposed preeclampsia were more likely to be nulliparous. However, the proportions of birth order 1 in women with chronic hypertension without preeclampsia and normotesive women were not significantly different. Women who smoked were younger than non-smokers.

Smokers had lower rates of transient hypertension and preeclampsia than nonsmokers (**Table 2 **[see Additional file 1]) with ORs between 0.7 and 0.8. However, the occurrence of chronic hypertension was statistically non-different in smokers and non-smokers.

**Figure 1:**
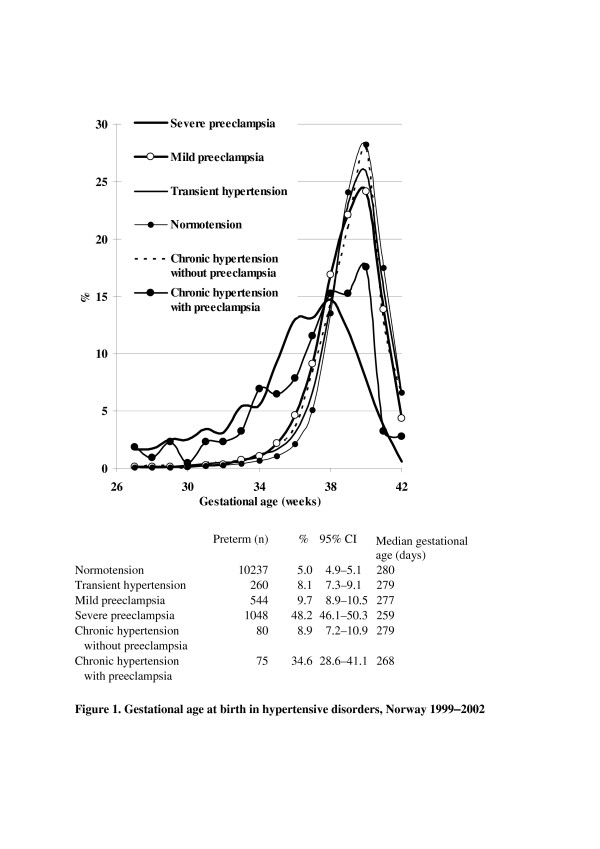


Mothers with severe preeclampsia or preeclampsia superimposed on chronic hypertension gave birth earlier than those who had mild preeclampsia or transient hypertension (Figure 1). Median gestational age at birth in transient hypertension, mild-, and severe preeclampsia were 279, 277, and 259 days, respectively, against 280 days in normotensive pregnancies, while those with chronic hypertension with and without preeclampsia had median gestational age of 268 and 279 days, respectively. The rates of preterm birth (less than 37 weeks of gestation) in transient hypertension, mild-, and severe preeclampsia were 8.1, 9.7, and 48.2%, respectively, against 5.0% in normotensive women. The rates were also significantly higher in chronic hypertension with or without preeclampsia (34.5% and 8.9%).

Birthweights were lower in smokers than in non-smokers (**Table 3 **[see Additional file 1]). For term births, mean birthweights in non-smokers consistently decreased with severity of PIH. Thus, in transient hypertension, mild- or severe preeclampsia mean birthweights were 3596 g, 3524 g, and 3274 g, respectively, against 3644 g for normotensive women. For smokers, no such trend was found. However, irrespective of smoking status and time of delivery, those with severe preeclampsia had lower birthweights than those with mild preeclampsia and transient hypertension, but without significant differences in smokers who delivered preterm.

In the assessment of the smoking effect, it appeared that in preterm and term births, smokers had consistently higher rates of SGA (**Table 4 **[see Additional file 1]) and reduction in birthweight (**Table 5 **[see Additional file 2]). Infants of normotensive women who smoked were twice as likely to be SGA (ORs 2.0 and 1.7 in term and preterm births, respectively (**Table 4 **[see Additional file 1]). Contrary to a hypothesis that the effect of smoking on fetal growth increases with severity of PIH, the effect on SGA rather seemed to decrease with severity (**Table 4 **[see Additional file 1]). Thus, in severe preeclampsia in term births, ORs of SGA in smokers compared with non-smokers was 1.4 compared with 2.0 in normotensive smokers. For preterm births, corresponding ORs were 1.3 and 1.7, respectively. However, neither in preterm- nor in term births, the ORs were significantly different. Consistently, in term births with transient hypertension, mild-, or severe preeclampsia, mean birthweight deficits attributable to smoking were 0.40 SD scores (171 g), 0.19 SD scores (64 g), and 0.18 SD scores (75 g), respectively (**Table 5 **[see Additional file 2]). For preterm births, corresponding deficits were 0.61 SD scores (320 g), 0.40 SD scores (327 g), and 0.13 SD scores (86 g).

In the assessment of the effect of PIH it appeared that in preterm and term births in smokers as well as non-smokers, women with PIH had consistently higher rates of SGA (**Table 4 **[see Additional file 1]). Women with PIH had excess birthweight deficit (**Table 5 **[see Additional file 2]), with the exception of smokers who delivered at term. In general, the effect of PIH, as indicated by ORs of SGA (**Table 4 **[see Additional file 1] and birthweight deficit (**Table 5 **[see Additional file 2]), were significantly more pronounced in those who delivered preterm than at term. Only in non-smokers who delivered at term, rates of SGA significantly increased with the severity of PIH (ORs = 1.3, 1.8, and 2.5 for transient hypertension, mild-, and severe preeclampsia, respectively) (**Table 4 **[see Additional file 1]). Consistently, birthweight deficits also significantly increased with the severity of PIH and were 0.03 SD scores (48 g), 0.07 SD scores (120 g), and 0.28 SD scores (370 g) for transient hypertension, mild-, and severe preeclampsia, respectively. In smokers a weaker, if any effect of severity of PIH was noted. Nor in preterm births, the smoking effects on SGA were significantly modified by severity of PIH.

The effect of smoking on birthweight was not significantly stronger in women who had chronic hypertension or superimposed preeclampsia than in normotensive women (**Table 4 **[see Additional file 1]). Nor were the effects of chronic hypertension with and without preeclampsia significantly different in smokers and non-smokers.

To test for significant interactions between smoking and hypertension, we added multiplicative interaction terms of smoking with each of the five hypertensive disorders in the logistic regression models for term and preterm births. In term births, the only significant interaction was mild preeclampsia × smoking (OR compared with normotensive non-smokers = 0.8; *p *= 0.016) and thus the actual OR for smokers with mild preeclampsia of 2.8 (**Table 4 **[see Additional file 1]) (1.8 × 2.0 × 0.8) would suggest antagonistic effects. In preterm births, transient hypertension × smoking (OR = 2.5; *p *= 0.024) was significant. Thus, the actual OR of 10.9 (2.6 × 1.7 × 2.5) (**Table 4 **[see Additional file 1]) in smokers with transient hypertension indicated synergistic effects.

For preterm-, term births, and the whole gestational age period, the sums of adjusted category specific population attributable risk fractions for SGA were 34.5, 16.6, and 17.7%, respectively (**Table 6 **[see Additional file 2]). Thus, 34.5% and 16.6% of all cases of SGA in the population born preterm and at term were attributable to the combined effects of PIH, chronic hypertension, smoking, and high maternal age (35 years+). PIH explained 21.9% (2.6 + 7.9 + 11.4) of preterm cases of SGA and 2.5% of term cases while chronic hypertension explained 1% or less. Smoking alone explained 9.3 and 11.9% of preterm and term SGA cases.

## Discussion

We found lower birthweights in infants of mothers who were smoking or had a hypertensive disorder in pregnancy. The combined effects of smoking and hypertension on birthweight were generally not synergistic. Our study indicates that the effect of early onset PIH was significantly stronger than the effect of late onset, while the effect of smoking was equal in preterm and term newborns. Smoking explained more than 9 and 12% of preterm and term cases of SGA, while hypertension explained 22% of SGA cases in preterm births and less than 2.5% in term births.

A strength of this study was its large size and the inclusion of data on subgroups of hypertensive disorders. Based on data from the total Norwegian birth population, the study was most likely not affected by selection bias. Data on maternal smoking were collected in early pregnancy, precluding recall bias. Some of the effect of PIH on birthweight might be explained by shared risk factors for PIH and FGR that were not adjusted for in the present analysis, such as thrombophilia which have been associated with both conditions [22], but not consistently [23]. However, most established risk factors for PIH and FGR, such as maternal short stature are weak or uncommon and would not influence the associations essentially. Due to lack of data, we were not able to include in the study excessive weight gain and prepregnancy weight, which are both strong and common risk factors for PIH, but tend to increase birthweight [24]. Thus, adjusting for maternal weight or weight gain would increase rather than decrease the effect of PIH on birthweight.

Consistent with earlier studies [11-13] that reported no significant interaction of smoking with PIH, the effect of smoking in the present study was not more pronounced in those who also had PIH. Although not significant, the effect of smoking in women with PIH even seemed to decrease with severity of PIH. Thus, the combination of preterm transient hypertension and smoking significantly exceeded the expected effect under a multiplicative model (Table 4). A Swedish study [9] reported much higher smoking-attributable fetal growth deficit in smokers who developed preeclampsia, which was consistent with a synergistic effect. The reason for the conflicting results with the Swedish study is obscure. In that study, SGA was defined as birthweight less than 2 SDs below the birthweight mean for gestational age, which corresponds to about the 2.5^th ^percentile, while we defined SGA as birthweight below the 10^th ^percentile (1.282 SD below the study population's mean curve for gestational age). However, this can hardly explain the discrepancy.

Few studies have compared the effects of smoking in infants born preterm and at term. In the present study, nomotensive smokers were twice more likely than normotensive non-smokers to deliver an SGA infant to term as well as preterm, which agrees with a Swedish study [25]. We are not aware of studies that have compared the effects of smoking in early and late onset subgroups of PIH. As in normotensive pregnancies, we found statistically equal effects of smoking in preterm- and term transient hypertension, mild-, and severe preeclampsia.

Our finding, that PIH was associated with decreased fetal growth, agrees with earlier studies [7,9,11,13]. Cnattingius *et al*. [9] reported increasing effect on fetal growth with severity of PIH both in smokers and non-smokers. In the present study, the effect of PIH increased significantly with severity of PIH only in non-smokers with late onset PIH. The effect of early onset PIH on fetal growth was significantly more pronounced than PIH in women who delivered to term, which is consistent with earlier studies that even report excess of large newborns in late onset PIH [6,7].

Smoking and PIH have been reported to exert their effects on fetal growth through different placental and vascular mechanisms. Clinical studies have suggested that in early onset preclampsia, FGR often precedes the development of hypertension and proteinuria [4]. Shallow invasion by fetal trophoblasts in maternal spiral arteries in early pregnancy, which may cause occlusion of the spiral arteries, has been observed both in preeclampsia and FGR [1,2]. Adaptive changes on the fetal side such as increased capillary diameter and proliferation of the trophoblastic villi have been considered secondary to the maternal vascular lesions [26]. In contrast to these adaptive changes, decreased fetal capillary diameter and no evidence of trophoblast proliferation have been found in smokers [27]. Eventually, products released by the ischemic placenta cause maternal endothelial activation which leads to preeclampsia [28,29]. Also this secondary endothelial activation involves several mechanisms that may affect fetal growth [29-32]. It is well known that smoking also leads to endothelial alterations. However, in pregnant women smoking has been associated with reduced markers of endothelial activation such as cellular fibronectin and vascular cell adhesion molecule-1, which is consistent with the reduced incidence of PIH in smokers [33,34].

In the present study, the effects of hypertensive disorder and smoking were generally not synergistic, which suggests that they may exert their main actions on separate sites or work through separate mechanisms within or outside the placenta [19]. Still, some of these pathways seem, at least to some extent, to be shared by smoking and PIH. However, the relative importance of each mechanism in smoking and PIH is unknown. Among vascular mechanisms are endothelial activation which likely causes reduced production or activity of vasodilators such as prostacyclin [30], nitric oxide [31], and increased levels of the potent vasoconstrictors thromboxane A2 [32] and endothelin-1 [29], thereby reducing utero-placental blood flow and consequently fetal growth. Smokers also have decreased nitric oxide synthetase activities in the placenta [35], raised endothelin-1 activity [36,37], and raised thomboxane to prostacyclin ratio, which may contribute to the reduced fetal growth, although smokers have raised prostacyclin as well as thromboxane activities [38,39]. Carbon monoxide from tobacco smoke interferes with oxygen transfer and replaces oxygen from maternal and fetal hemoglobin and thus reduces fetal growth [40], but is unclear whether carbon monoxide is involved in the effects of hypertension on fetal growth.

Of interest is that 11% of preterm SGA cases were explained by severe preeclampsia, while chronic hypertension explained 1%. In the present study, PIH explained 2.5% and 22% of all term and preterm SGA cases, respectively. Our results confirm that smoking is an important cause of FGR. Smoking, which is regarded as the most important known preventable risk factor for FGR, explained about 12% SGA cases which agrees with other recent studies [41,42]. This means that the number of SGA cases would be reduced by 12%, or about 2600 cases in the study population, if smoking were eliminated in pregnant women.

## Conclusion

The effects of hypertensive disorder and smoking are generally not synergistic, which suggest that they may exert their main actions on separate sites or work through separate mechanisms within or outside the placenta. Infants of mothers who smoke or have a hypertensive disorder in pregnancy have reduced birthweights. The effect of early onset PIH seems to be significantly stronger than the effect of late onset, while the effect of smoking is equal in preterm and term newborns. If smoking were eliminated in pregnant women, the number of SGA cases would be reduced by 12%, while hypertension explained 22% of SGA cases in preterm births and less than 2.5% in term births.

## Competing interests

The author(s) declare that the have no competing interests

## Authors' contributions

SR had the original idea, carried out the analyses, drafted and edited the paper.

LMI contributed on the design of the study and writing of the manuscript. Both authors read and approved the final manuscript.

## Pre-publication history

The pre-publication history for this paper can be accessed here:



## Supplementary Material

Additional file 1 **Table 1 **– Births by smoking status, maternal age, and birth order in normotension and hypertensive disorders in Norway 1999–2002. **Table 2 **– Proportions and odds ratios (OR) (95% confidence interval (CI)) of hypertensive disorders in smokers compared with non-smokers delivering preterm, at term, and for the whole period of gestation, adjusted for maternal age and birth order, Norway 1999–2002. **Table 3 **– Unadjusted mean birthweight in normotension and hypertensive disorders in term- and preterm births by maternal smoking habits, Norway 1999–2002. **Table 4 **– Occurrence of small for gestational age (below the 10th birthweight percentile) in term- and preterm births according to maternal hypertension and smoking status. Odds ratios (OR) (95% confidence interval (CI)) compared with normotension, and non-smokers, adjusted for maternal, Norway 1999–2002.
Click here for file

Additional file 2 **Table 5 **– Mean reduction in standard deviation score in grams of birthweight attributable to smoking and hypertensive disorders in term- and preterm births, adjusted for maternal age, Norway 1999–2002. **Table 6 **– Population attributable risk fractions of small for gestational age (birthweight below the 10th percentile), due to smoking and hypertensive disorders in preterm-, term births, and for the total period of gestation in Norway 1999–2002.
Click here for file
