# Supplementary material for: The effects of smoking and hypertensive disorders on fetal growth
Source: BMC Pregnancy Childbirth. 2006 Apr 21;6:16. doi: 10.1186/1471-2393-6-16 (PMC1463005; doi:10.1186/1471-2393-6-16)
Supplement: Additional file 1 — Table 1 – Births by smoking status, maternal age, and birth order in normotension and hypertensive disorders in Norway 1999–2002. Table 2 – Proportions and odds ratios (OR) (95% confidence interval (CI)) of hypertensive disorders in smokers compared with non-smokers delivering preterm, at term, and for the whole period of gestation, adjusted for maternal age and birth order, Norway 1999–2002. Table 3 – Unadjusted mean birthweight in normotension and hypertensive disorders in term- and preterm births by maternal smoking habits, Norway 1999–2002. Table 4 – Occurrence of small for gestational age (below the 10th birthweight percentile) in term- and preterm births according to maternal hypertension and smoking status. Odds ratios (OR) (95% confidence interval (CI)) compared with normotension, and non-smokers, adjusted for maternal, Norway 1999–2002. [file 1471-2393-6-16-S1.doc]

| **Table 1. Births by smoking status, maternal age, and birth order in normotension and hypertensive disorders in Norway 1999–2002** | | | | | | | | | | | | | | | | | | | | | | | | |  | |  | | |
| --- | --- | --- | --- | --- | --- | --- | --- | --- | --- | --- | --- | --- | --- | --- | --- | --- | --- | --- | --- | --- | --- | --- | --- | --- | --- | --- | --- | --- | --- |
|  |  | Total |  |  | Smoker |  |  |  |  | Maternal age in years | | | | |  | |  | |  | |  | Birth order 1 | | | | | |  | |
|  |  | *n* | % |  | *n* | % | 95% CI | | |  | Mean | 95% CI | | 35 years + | | % | | 95% CI | | | |  | *n* | % | | 95% CI | | | |
| Hypertensive disorder: | |  |  |  |  |  |  |  | |  |  |  |  |  | |  | |  | |  | |  |  |  | |  | | |  |
|  | Normotension | 203498 | 94.4 |  | 34387 | 16.9 | [17, | 17] | |  | 29.1 | [29.1, | 29.1] | 29135 | | 14.3 | | [14.2, | | 14.5] | |  | 83744 | 41.2 | | [40.9, | | | 41.4] |
|  | Transient hypertension | 3191 | 1.5 |  | 387 | 12.1 | [11, | 13] | |  | 29.9 | [29.7, | 30.1] | 580 | | 18.2 | | [16.9, | | 19.6] | |  | 1626 | 51.0 | | [49.2, | | | 52.7] |
|  | Mild preeclampsia | 5614 | 2.6 |  | 758 | 13.5 | [13, | 14] | |  | 28.7 | [28.5, | 28.8] | 763 | | 13.6 | | [12.7, | | 14.5] | |  | 3358 | 59.8 | | [58.5, | | | 61.1] |
|  | Severe preeclampsia | 2176 | 1.0 |  | 260 | 11.9 | [11, | 13] | |  | 28.3 | [28.1, | 28.5] | 265 | | 12.2 | | [10.9, | | 13.6] | |  | 1455 | 66.9 | | [64.9, | | | 68.8] |
|  | Chronic hypertension | 902 | 0.4 |  | 151 | 16.7 | [15, | 19] | |  | 30.8 | [30.5, | 31.1] | 234 | | 25.9 | | [23.2, | | 28.9] | |  | 308 | 34.1 | | [31.1, | | | 37.3] |
|  | without preeclampsia |  |  |  |  |  |  |  | |  |  |  |  |  | |  | |  | |  | |  |  |  | |  | | |  |
|  | Chronic hypertension | 217 | 0.1 |  | 29 | 13.4 | [10, | 19] | |  | 31.1 | [30.4, | 31.7] | 59 | | 27.2 | | [21.7, | | 33.5] | |  | 99 | 45.6 | | [39.1, | | | 52.3] |
|  | with preeclampsia |  |  |  |  |  |  |  | |  |  |  |  |  | |  | |  | |  | |  |  |  | |  | | |  |
| Smoking status: | |  |  |  |  |  |  |  | |  |  |  |  |  | |  | |  | |  | |  |  |  | |  | | |  |
|  | Non-smoker | 179626 | 83.3 |  |  |  |  |  | |  | 29.3 | [29.3, | 29.3] | 26466 | | 14.7 | | [14.6, | | 14.9] | |  | 75267 | 41.9 | | [41.9, | | | 42.1] |
|  | Smoker | 35972 | 16.7 |  |  |  |  |  | |  | 28.1 | [28.0, | 28.1] | 4570 | | 12.7 | | [12.4, | | 13.1] | |  | 15323 | 42.6 | | [42.1, | | | 43.1] |

CI: confidence interval

| **Table 2. Proportions and odds ratios (OR) (95% confidence interval (CI)) of hypertensive disorders in smokers compared with non-smokers delivering preterm, at term, and for the whole period of gestation, adjusted for maternal age and birth order, Norway 1999–2002** | | | | | | | | | | | | | | | | | | |
| --- | --- | --- | --- | --- | --- | --- | --- | --- | --- | --- | --- | --- | --- | --- | --- | --- | --- | --- |
|  |  | **Preterm** |  |  |  |  |  | **Term** |  |  |  |  |  | **Total** |  |  |  |  |
|  |  | **N** | **%** | **OR** | **95% CI** | |  | **N** | **%** | **OR** | **95% CI** | |  | **N** | **%** | **OR** | **95% CI** | |
| **Non-smokers** | |  |  |  |  |  |  |  |  |  |  |  |  |  |  |  |  |  |
|  | Normotensive | 8111 | 82.3 |  |  |  |  | 161000 | 94.8 |  |  |  |  | 169111 | 94.1 |  |  |  |
|  | Transient hypertension | 228 | 2.3 | 1 |  |  |  | 2576 | 1.5 | 1 |  |  |  | 2804 | 1.6 | 1 |  |  |
|  | Mild preeclampsia | 468 | 4.7 | 1 |  |  |  | 4388 | 2.6 | 1 |  |  |  | 4856 | 2.7 | 1 |  |  |
|  | Severe preeclampsia | 918 | 9.3 | 1 |  |  |  | 998 | 0.6 | 1 |  |  |  | 1916 | 1.1 | 1 |  |  |
|  | Chronic hypertension without | 67 | 0.7 | 1 |  |  |  | 684 | 0.4 | 1 |  |  |  | 751 | 0.4 | 1 |  |  |
|  | preeclampsia |  |  |  |  |  |  |  |  |  |  |  |  |  |  |  |  |  |
|  | Chronic hypertension with | 62 | 0.6 | 1 |  |  |  | 126 | 0.1 | 1 |  |  |  | 188 | 0.1 | 1 |  |  |
|  | preeclampsia |  |  |  |  |  |  |  |  |  |  |  |  |  |  |  |  |  |
| **Smokers** | |  |  |  |  |  |  |  |  |  |  |  |  |  |  |  |  |  |
|  | Normotensive | 2126 | 89.0 |  |  |  |  | 32261 | 96.1 |  |  |  |  | 34387 | 95.6 |  |  |  |
|  | Transient hypertension | 32 | 1.3 | 0.60 | 0.41 | 0.87 |  | 355 | 1.1 | 0.71 | 0.64 | 0.80 |  | 387 | 1.1 | 0.70 | 0.63 | 0.78 |
|  | Mild preeclampsia | 76 | 3.2 | 0.66 | 0.52 | 0.85 |  | 682 | 2.0 | 0.78 | 0.72 | 0.85 |  | 758 | 2.1 | 0.78 | 0.72 | 0.84 |
|  | Severe preeclampsia | 130 | 5.4 | 0.57 | 0.47 | 0.69 |  | 130 | 0.4 | 0.65 | 0.54 | 0.78 |  | 260 | 0.7 | 0.67 | 0.59 | 0.76 |
|  | Chronic hypertension without | 13 | 0.5 | 0.93 | 0.51 | 1.69 |  | 138 | 0.4 | 0.67 | 0.40 | 1.13 |  | 151 | 0.4 | 0.81 | 0.55 | 1.20 |
|  | preeclampsia |  |  |  |  |  |  |  |  |  |  |  |  |  |  |  |  |  |
|  | Chronic hypertension with | 13 | 0.5 | 0.86 | 0.47 | 1.57 |  | 16 | 0.0 | 1.07 | 0.89 | 1.28 |  | 29 | 0.1 | 1.05 | 0.88 | 1.26 |
|  | preeclampsia |  |  |  |  |  |  |  |  |  |  |  |  |  |  |  |  |  |

| **Table 3. Unadjusted mean birthweight in normotension and hypertensive disorders in term- and preterm births by maternal smoking habits, Norway 1999–2002** | | | | | |
| --- | --- | --- | --- | --- | --- |
|  | Preterm |  | Term |  | Total |
|  | Mean |  | Mean |  | Mean |
| **Non smokers** |  |  |  |  |  |
| Normotension | 2472 |  | 3644 |  | 3577 |
| Transient hypertension | 2263 |  | 3596 |  | 3463 |
| Mild preeclampsia | 2355 |  | 3524 |  | 3390 |
| Severe preeclampsia | 1923 |  | 3274 |  | 2537 |
| Chronic hypertension without preeclampsia | 2184 |  | 3574 |  | 3420 |
| Chronic hypertension with preeclampsia | 2046 |  | 3366 |  | 2857 |
| **Smokers** |  |  |  |  |  |
| Normotension | 2350 |  | 3496 |  | 3411 |
| Transient hypertension | 1943 |  | 3425 |  | 3268 |
| Mild preeclampsia | 2028 |  | 3460 |  | 3279 |
| Severe preeclampsia | 1837 |  | 3200 |  | 2425 |
| Chronic hypertension without preeclampsia | 2387 |  | 3489 |  | 3377 |
| Chronic hypertension with preeclampsia | 1827 |  | 3474 |  | 2605 |

| **Table 4. Occurrence of small for gestational age (below the 10th birthweight percentile) in term- and preterm births according to maternal hypertension and smoking status. Odds ratios (OR) (95% confidence interval (CI)) compared with normotension, and non-smokers, adjusted for maternal, Norway 1999–2002** | | | | | | | | | | | | | | | | | |
| --- | --- | --- | --- | --- | --- | --- | --- | --- | --- | --- | --- | --- | --- | --- | --- | --- | --- |
|  |  | | <10th birthweight percentile | | | | | | | | | | | | | | |
|  |  |  |  | | | | | |  | | Effect of smoking | | |  | Effect of hypertension | | |
|  |  | | *N* | % | OR 95% CI | | |  | | OR 95% CI | | | |  | OR 95% CI | | |
| **Term** |  | |  |  |  |  |  |  | |  | |  |  |  |  |  |  |
| **Non smokers** |  | |  |  |  |  |  |  | |  | |  |  |  |  |  |  |
| Normotension |  | | 13550 | 8.4 | 1 |  |  |  | | 1 | |  |  |  | 1 |  |  |
| Transient hypertension | | 273 | | 10.6 | 1.3 | [1.1, | 1.5] |  | | 1 | |  |  |  | 1.3 | [1.1 | 1.5] |
| Mild preeclampsia | | 625 | | 14.2 | 1.8 | [1.7, | 2.0] |  | | 1 | |  |  |  | 1.8 | [1.7 | 2.0] |
| Severe preeclampsia | | 188 | | 18.8 | 2.5 | [2.2, | 3.0] |  | | 1 | |  |  |  | 2.5 | [2.2 | 3.0] |
| Chronic hypertension without preeclampsia | | 82 | | 12.0 | 1.5 | [1.2, | 1.9] |  | | 1 | |  |  |  | 1.5 | [1.2 | 1.9] |
| Chronic hypertension with preeclampsia | | 20 | | 15.9 | 2.1 | [1.3, | 3.3] |  | | 1 | |  |  |  | 2.1 | [1.3 | 3.3] |
| **Smokers** |  | |  |  |  |  |  |  | |  | |  |  |  |  |  |  |
| Normotension |  | | 5018 | 15.6 | 2.0 | [1.9, | 2.1] |  | | 2.0 | | [1.9, | 2.1] |  | 1 |  |  |
| Transient hypertension | | 77 | | 21.7 | 3.0 | [2.3, | 3.9] |  | | 2.3 | | [1.8, | 3.1] |  | 1.5 | [1.2, | 1.9] |
| Mild preeclampsia | | 140 | | 20.5 | **2.8*** | [2.3, | 3.4] |  | | 1.6 | | [1.3, | 1.9] |  | 1.4 | [1.2, | 1.7] |
| Severe preeclampsia | | 32 | | 24.6 | 3.6 | [2.4, | 5.3] |  | | 1.4 | | [0.9, | 2.2] |  | 1.8 | [1.2, | 2.6] |
| Chronic hypertension without preeclampsia | | 25 | | 18.1 | 2.4 | [1.6, | 3.7] |  | | 1.6 | | [1.0, | 2.7] |  | 1.2 | [0.8, | 1.9] |
| Chronic hypertension with preeclampsia | | 0 | |  |  |  |  |  | |  | |  |  |  |  |  |  |
| **Preterm** |  | |  |  |  |  |  |  | |  | |  |  |  |  |  |  |
| **Non smokers** |  | |  |  |  |  |  |  | |  | |  |  |  |  |  |  |
| Normotension |  | | 605 | 7.5 | 1 |  |  |  | | 1 | |  |  |  | 1 |  |  |
| Transient hypertension | | 40 | | 17.5 | 2.6 | [1.9, | 3.8] |  | | 1 | |  |  |  | 2.6 | [1.9, | 3.8] |
| Mild preeclampsia | | 119 | | 25.4 | 4.2 | [3.4, | 5.3] |  | | 1 | |  |  |  | 4.2 | [3.4, | 5.3] |
| Severe preeclampsia | | 199 | | 21.7 | 3.4 | [2.9, | 4.1] |  | | 1 | |  |  |  | 3.4 | [2.9, | 4.1] |
| Chronic hypertension without preeclampsia | | 12 | | 17.9 | 2.7 | [1.4, | 5.1] |  | | 1 | |  |  |  | 2.7 | [1.4, | 5.1] |
| Chronic hypertension with preeclampsia | | 8 | | 12.9 | 1.8 | [0.9, | 3.9] |  | | 1 | |  |  |  | 1.8 | [0.9, | 3.9] |
| **Smokers** |  | |  |  |  |  |  |  | |  | |  |  |  |  |  |  |
| Normotension |  | | 253 | 11.9 | 1.7 | [1.4, | 2.0] |  | | 1.7 | | [1.4, | 2.0] |  | 1 |  |  |
| Transient hypertension | | 15 | | 46.9 | **10.9*** | [5.4, | 22.0 |  | | 4.1 | | [1.9, | 9.0] |  | 6.5 | [3.2, | 13.2] |
| Mild preeclampsia | | 29 | | 38.2 | 7.7 | [4.8, | 12.3 |  | | 1.8 | | [1.1, | 3.0] |  | 4.6 | [2.8, | 7.4] |
| Severe preeclampsia | | 35 | | 26.9 | 4.6 | [3.1, | 6.8 |  | | 1.3 | | [0.9, | 2.0] |  | 2.7 | [1.8, | 4.1] |
| Chronic hypertension without preeclampsia | | 3 | | 23.1 | 3.7 | [1.0, | 13.6 |  | | 1.4 | | [0.3, | 5.8] |  | 2.2 | [0.6, | 8.1] |
| Chronic hypertension with preeclampsia | | 3 | | 23.1 | 3.7 | [1.0, | 13.6 |  | | 2.0 | | [0.5, | 9.0] |  | 2.2 | [0.6, | 8.1] |
| ***** Significant interaction between hypertension and smoking status | | | | | | | | | | | | | | | | | |
