# Supplementary material for: The effects of smoking and hypertensive disorders on fetal growth
Source: BMC Pregnancy Childbirth. 2006 Apr 21;6:16. doi: 10.1186/1471-2393-6-16 (PMC1463005; doi:10.1186/1471-2393-6-16)
Supplement: Additional file 2 — Table 5 – Mean reduction in standard deviation score in grams of birthweight attributable to smoking and hypertensive disorders in term- and preterm births, adjusted for maternal age, Norway 1999–2002. Table 6 – Population attributable risk fractions of small for gestational age (birthweight below the 10th percentile), due to smoking and hypertensive disorders in preterm-, term births, and for the total period of gestation in Norway 1999–2002. [file 1471-2393-6-16-S2.doc]

| **Table 5. Mean reduction in standard deviation score in grams of birthweight attributable to smoking and hypertensive disorders in term- and preterm births, adjusted for maternal age, Norway 1999–2002** | | | | | | | | |
| --- | --- | --- | --- | --- | --- | --- | --- | --- |
|  |  | Mean reduction of standard deviation score attributable to | | | | | | |
| **Term** |  | Smoking | 95% CI | |  | Hypertension | 95% CI | |
|  |  |  |  |  |  |  |  |  |
| **Non smokers** |  |  |  |  |  |  |  |  |
| Normotension |  | 0 |  |  |  | 0 |  |  |
| Transient hypertension |  | 0 |  |  |  | 0.03 | [-0.01, | 0.07] |
| Mild preeclampsia |  | 0 |  |  |  | 0.07 | [0.04, | 0.10] |
| Severe preeclampsia |  | 0 |  |  |  | 0.28 | [0.21, | 0.34] |
| Chronic hypertension without preeclampsia |  | 0 |  |  |  | 0.10 | [0.02, | 0.18] |
| Chronic hypertension with preeclampsia |  | 0 |  |  |  | 0.31 | [0.13, | 0.49] |
| **Smokers** |  |  |  |  |  |  |  |  |
| Normotension |  | 0.31 | [0.30, | 0.33] |  | 0 |  |  |
| Transient hypertension |  | 0.40 | [0.28, | 0.51] |  | 0.12 | [0.01, | 0.22] |
| Mild preeclampsia |  | 0.19 | [0.10, | 0.27] |  | -0.06 | [-0.13, | 0.02] |
| Severe preeclampsia |  | 0.18 | [-0.01, | 0.38] |  | 0.15 | [-0.03, | 0.33] |
| Chronic hypertension without preeclampsia |  | 0.18 | [-0.01, | 0.37] |  | -0.03 | [-0.20, | 0.15] |
| Chronic hypertension with preeclampsia |  | -0.38 | [-0.92, | 0.16] |  | -0.39 | [-0.90, | 0.12] |
|  |  |  |  |  |  |  |  |  |
| **Preterm** |  |  |  |  |  |  |  |  |
| **Non smokers** |  |  |  |  |  |  |  |  |
| Normotension |  | 0 |  |  |  | 0 |  |  |
| Transient hypertension |  | 0 |  |  |  | 0.46 | [0.31, | 0.61] |
| Mild preeclampsia |  | 0 |  |  |  | 0.56 | [0.45, | 0.66] |
| Severe preeclampsia |  | 0 |  |  |  | 0.74 | [0.67, | 0.82] |
| Chronic hypertension without preeclampsia |  | 0 |  |  |  | 0.58 | [0.31, | 0.85] |
| Chronic hypertension with preeclampsia |  | 0 |  |  |  | 0.43 | [0.15, | 0.71] |
| **Smokers** |  |  |  |  |  |  |  |  |
| Normotension |  | 0.26 | [0.21, | 0.31] |  | 0 |  |  |
| Transient hypertension |  | 0.61 | [0.20, | 1.02] |  | 0.81 | [0.42, | 1.20] |
| Mild preeclampsia |  | 0.40 | [0.13, | 0.67] |  | 0.69 | [0.44, | 0.95] |
| Severe preeclampsia |  | 0.13 | [-0.07, | 0.34] |  | 0.61 | [0.41, | 0.81] |
| Chronic hypertension without preeclampsia |  | 0.19 | [-0.47, | 0.86] |  | 0.51 | [-0.10, | 1.12] |

| **Table 6. Population attributable risk fractions of small for gestational age (birthweight below the 10th percentile), due to smoking and hypertensive disorders in preterm-, term births, and for the total period of gestation in Norway 1999–2002** | | | | | | | |
| --- | --- | --- | --- | --- | --- | --- | --- |
|
|
|  | Adjusted* population attributable | | | | | | |
|  | risk fraction (%) | | | | | | |
|  | Preterm birth | |  | | Term birth |  | Total |
|  |  |  | |  | |  |  |
| **Pregnancy induced** |  |  | |  | |  |  |
| **Hypertension** |  |  | |  | |  |  |
| Transient hypertension | 2.6 |  | | 0.4 | |  | 0.5 |
| Mild preeclampsia | 7.9 |  | | 1.5 | |  | 1.8 |
| Severe preeclampsia | 11.4 |  | | 0.6 | |  | 1.2 |
|  |  |  | |  | |  |  |
| **Chronic hypertension** |  |  | |  | |  |  |
| with preeclampsia | 0.6 |  | | 0.1 | |  | 0.2 |
| without preeclampsia | 0.4 |  | | 0.03 | |  | 0.05 |
|  |  |  | |  | |  |  |
| **Smoking** | 9.3 |  | | 11.9 | |  | 11.8 |
|  |  |  | |  | |  |  |
| **Maternal age (years)** |  |  | |  | |  |  |
| 35 years+ | 2.3 |  | | 2.1 | |  | 2.1 |
|  |  |  | |  | |  |  |
| **Sum of adjusted** | 34.5 |  | | 16.6 | |  | 17.7 |
| **category specific** |  |  | |  | |  |  |
| **attributable risk fractions** |  |  | |  | |  |  |
| * Adjusted for maternal age and smoking | | | | | | | |
